# GPR Expression in Intestinal Biopsies From SCT Patients Is Upregulated in GvHD and Is Suppressed by Broad-Spectrum Antibiotics

**DOI:** 10.3389/fimmu.2021.753287

**Published:** 2021-10-28

**Authors:** Sakhila Ghimire, Daniela Weber, Katrin Hippe, Elisabeth Meedt, Matthias Hoepting, Anna-Sophia Kattner, Andreas Hiergeist, André Gessner, Carina Matos, Saroj Ghimire, Daniel Wolff, Matthias Edinger, Petra Hoffmann, Hendrik Poeck, Wolfgang Herr, Ernst Holler

**Affiliations:** ^1^ Department of Internal Medicine III, University Hospital Regensburg, Regensburg, Germany; ^2^ Department of Pathology, University of Regensburg, Regensburg, Germany; ^3^ Institute for Medical Microbiology and Hygiene (IMHR), University Hospital Regensburg, Regensburg, Germany; ^4^ Kathmandu University School of Medical Sciences, Dhulikhel, Nepal; ^5^ Regensburg Center for Interventional Immunology (RCI), Regensburg, Germany

**Keywords:** broad-spectrum antibiotics, GPR, Foxp3, GvHD, microbiota, SCFA

## Abstract

Microbiota can exert immunomodulatory effects by short-chain fatty acids (SCFA) in experimental models of graft-versus-host disease (GvHD) after allogeneic hematopoietic stem cell transplantation (allo-SCT). Therefore we aimed to analyze the expression of SCFAs sensing G-protein coupled receptor GPR109A and GPR43 by quantitative PCR in 338 gastrointestinal (GI) biopsies obtained from 199 adult patients undergoing allo-SCT and assessed the interaction of GPR with FOXP3 expression and regulatory T cell infiltrates. GPR expression was strongly upregulated in patients with stage II-IV GvHD (p=0.000 for GPR109A, p=0.01 for GPR43) and at the onset of GvHD (p 0.000 for GPR109A, p=0.006 for GPR43) and correlated strongly with FOXP3 and NLRP3 expression. The use of broad-spectrum antibiotics (Abx) drastically suppressed GPR expression as well as FOXP3 expression in patients’ gut biopsies (p=0.000 for GPRs, FOXP3 mRNA and FOXP3+ cellular infiltrates). Logistic regression analysis revealed treatment with Abx as an independent factor associated with GPR and FOXP3 loss. The upregulation of GPRs was evident only in the absence of Abx (p=0.001 for GPR109A, p=0.014 for GPR43) at GvHD onset. Thus, GPR expression seems to be upregulated in the presence of commensal bacteria and associates with infiltration of FOXP3+ T regs, suggesting a protective, regenerative immunomodulatory response. However, Abx, which has been shown to induce dysbiosis, interferes with this protective response.

**Graphical Abstract:**
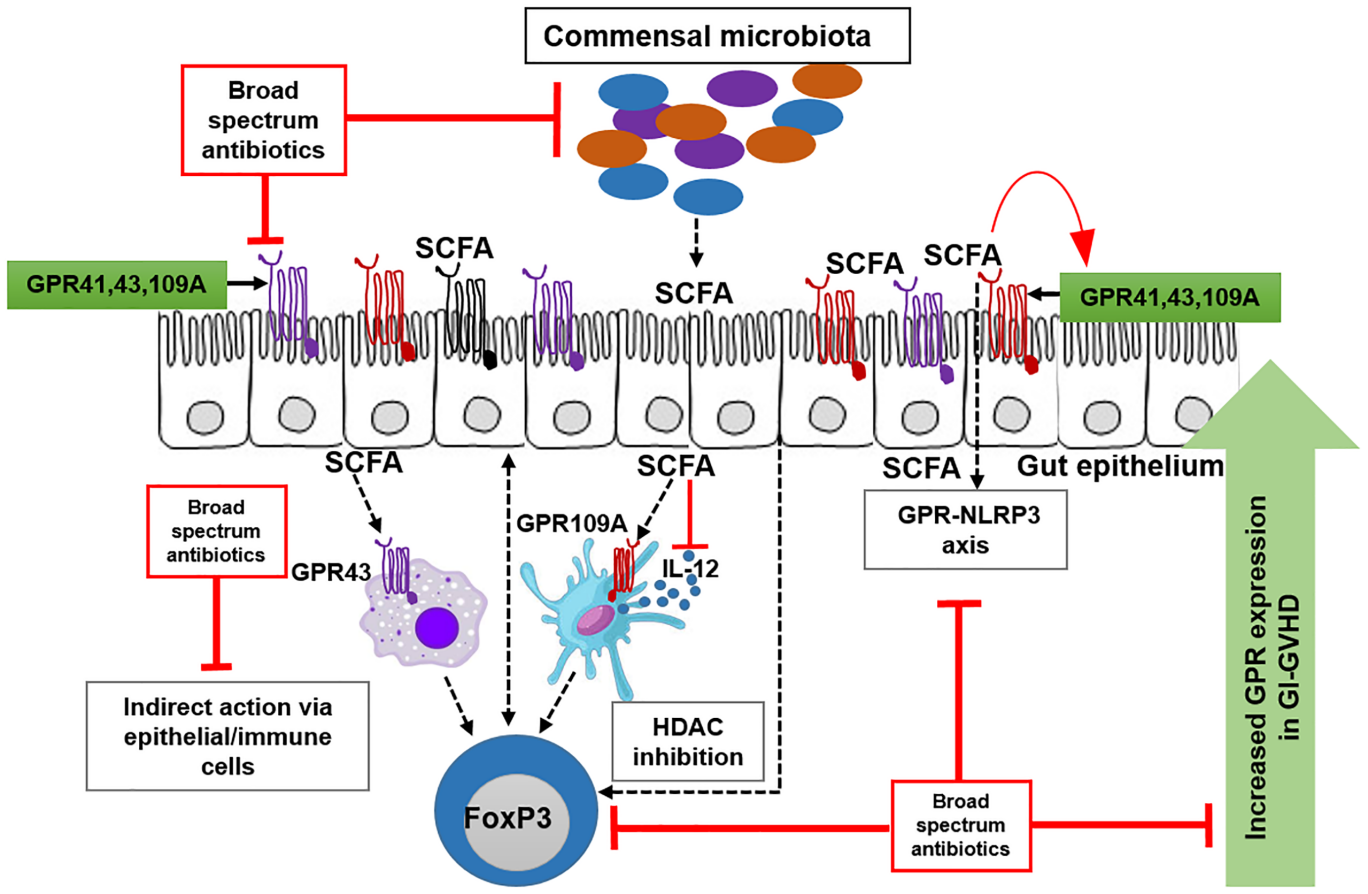
G-protein coupled receptor (GPR109A, GPR43 and GPR41) is predominantly expressed on epithelial and immune cells. GPR is activated by its ligand short chain fatty acids (SCFAs). In absence of broad-spectrum antibiotics (Abx), beneficial commensals produce SCFAs that activate GPR pathway. SCFA engage GPR-NLRP3 pathway for the maintainance of epithelial barrier. SCFA also engage GPR on immune cells to induce regulatory T cells. Patients who do not receive Abx show an upregulation of GPR expression in the presence of aGvHD suggesting a counterregulatory mechanism. Abx suppresses commensals leading to reduced SCFA hence less GPR. GPR-NLRP3 axis and GPR-Tregs axis are strongly abrogated by Abx. Abx also interfere with the upregulation of GPR during aGvHD.

## Introduction

Acute Graft versus host disease (aGvHD) is the major cause of transplant-related mortality (TRM) and morbidity following allogeneic stem cell transplantation (SCT). Current treatment options for this complication are poor if initial treatment with steroids has failed (1). Landmark studies in the early 70s by van Bekkum already pointed to a role of the intestinal microflora in gastrointestinal (GI) aGvHD (2) and suggested protection of germfree mice from GvHD. Preclinical and clinical studies therefore introduced prophylactic use of decontamination as an approach to reduce GvHD (3) and together with the concept of prevention of neutropenic gram-negative infections, antibiotic prophylaxis has become standard of care (4) With the introduction of next-generation sequencing technologies including 16s rRNA, it now became evident that the intestinal microbiota is an important modulator of aGvHD. Since 2012, several studies using this technique in experimental (5) and clinical settings (6, 7) reported a strong loss of commensal bacteria (dysbiosis) but no complete decontamination and an association of dysbiosis with the occurrence of GI aGvHD as well as several severe infectious complications following allogeneic SCT. Prophylactic and therapeutic antibiotics were even identified as the major driver of dysbiosis (8, 9) and these findings more and more questioned at least prophylactic concepts. Recent reports also suggested that even the reconstitution of commensal bacteria by fecal microbiota transfer (FMT) contributes favorably to the treatment of patients (pts) with steroid-refractory aGvHD (10–12).

The mechanisms of how commensal intestinal microbiota dampens intestinal inflammation in general and in the setting of aGvHD are still poorly understood. Microbial metabolites that are produced by commensal bacteria after digestion of dietary fibers, tryptophan, and other sources have been identified as major protective molecules that act as mediators of pathogen-host interaction and exert protective functions. In this context, short-chain fatty acids (SCFA) like butyrate and propionate are not only a major energy source for colonocytes but also stabilize the epithelium and dampen immune reactions by multiple mechanisms including regulation of Nlrp3-inflammasome dependent inflammation (13) and by induction of regulatory T cells (T regs) (14, 15). Indoles derived from dietary tryptophan stabilize the epithelium *via* induction of interleukin 22 in innate lymphoid cells and modulate inflammation by inducing anti-inflammatory cytokines such as interleukin 10 (16, 17). Strong protection against aGvHD by the tryptophan-metabolite Indol-3-carboxaldehyde (ICA) was observed in Swimm´s study (18) as gavage with ICA reduced aGvHD mortality to a large extent in a type I Interferon (IFN-I) dependent manner while maintaining graft-versus-leukemia activity.

As all these mechanisms have been reported to modify GvHD, it is not surprising that experimental reports found significant protection from GvHD by these metabolites. Mathewson and colleagues applied butyrate gavage and a cocktail of commensal clostridia known to be high SCFA producers in a murine model of GvHD and reported strong protection (19). Recently, the same group addressed the role of SCFA by using knockout mice for one of the receptors of SCFA, G-protein coupled receptor (GPR) 43, and reported that GPR43 knockout on non-hematopoietic cells led to accelerated and increased GvHD related mortality (20).

In humans, a comparable role of SCFA is likely and suggested by a recent analysis of Romick-Rosendale et al. (21) who reported reduced stool SCFAs after exposure to Abx suppressing commensals in children receiving HSCT but so far no data have been reported regarding the expression of GPR in adult human GvHD. We, therefore, performed an analysis of expression of the major SCFA receptors GPR43 and GPR109A by quantitative PCR in intestinal biopsies obtained from pts receiving allogeneic SCT at our unit. We observed upregulation of GPR in aGvHD which was strongly suppressed by broad-spectrum antibiotics.

## Material and Methods

### Patient Characteristics

338 serial biopsies were obtained and analyzed from a total of 199 adult patients (pts) receiving allogeneic SCT between Nov 2008 and Nov 2015. Patient characteristics are summarized in **Supplementary Table 1**. The disease status was defined according to the EBMT score (22). All pts gave informed consent, the biopsy studies and scientific analyses were approved by the local ethical review board (approval no 02/220 and 09/059). All studies were performed in accordance with the regulations of Helsinki. Serial biopsies were either obtained i) in the course of a screening study in asymptomatic, clinically aGvHD free pts or ii) because of clinical symptoms indicative of *de novo* onset or iii) persistence or recurrence of GI aGvHD. Biopsies were obtained through upper or lower GI endoscopy.

### Quantitative Real-Time PCR (qPCR)

qPCR on intestinal biopsies was performed according to RNA availability. 338 serial biopsies for *GPR109A*, 263 biopsies for *GPR43*, 103 biopsies for *NLRP3*, 281 biopsies for *FOXP3* mRNA and 240 biopsies for FOXP3 immunohistochemistry were available. Intestinal biopsies were immediately transferred to 500 µl RNA later (QIAGEN) and were stored at -80°C until RNA extraction. RNA was extracted using RNeasy Mini Kit (QIAGEN) as per manufacturer’s recommendation. RNA concentration and purity was monitored by NanoDrop and Bioanalyzer respectively. 1 µg of RNA was reverse transcribed to cDNA using *moloney murine leukemia virus* reverse transcriptase (Promega) following the manufacturer’s instructions. qPCR was performed on a Mastercycler Ep Realplex (Eppendorf) using QuantiFast SYBR Green PCR Kit (QIAGEN). *18S* ribosomal RNA was used as reference gene. Gene of interest was normalized to the reference gene.

Gene-specific primer sequences are as follows: *GPR109A*, forward: 5’ GCG-TTG-GGA-CTG-GAA-GTT-TG-3’, reverse: 5’- GCG-GTT-CAT-AGC-CAA-CAT-GA-3’; *GPR43*, forward: 5’- GTA-GCT-AAC-ACA-AGT-CCA-GTC-CT -3’, reverse: 5- CTA-GGT-GTT-GCT-TTG-AAG-CTT-GT -3’; *FOXP3*, forward: 5’-GAA-ACA-GCA-CAT-TCC-CAG-AGT-TC -3’; reverse: 5’- ATG-GCC-CAG-CGG-ATG-AG-3’; *NLRP3*, forward: 5’-GGA-CTG-AAG-CAC-CTG-TTG-TGC-A-3’, reverse: 5’- TCC-TGA-GTC-TCC-CAA-GGC-ATT-C-3’; *18S*, forward: 5’-ACC-GAT-TGG-ATG-GTT-TAG-TGA-G-3’, reverse: 5’-CCT-ACG-GAA-ACC-TTG-TTA-CGA-C-3’.

### Immunohistological Analysis

The same pathologist blinded to the clinical data assessed serial biopsies. GI-aGvHD was graded according to the Lerner grading system (23). The number of FOXP3 positive cells was determined by immunohistochemistry, analyzed with a Zeiss Axioskop 40 microscope. 2-3µm thick slides sectioned from the formalin-fixed and paraffin-embedded (FFPE) biopsies were deparaffined and stained automatically (Ventana Benchmark Ultra). After pre-treatment with CC1 buffer the immunohistochemical staining was performed with a monoclonal mouse antibody (1:120, eBioscience 14-4777, clone 236A/E7) and OptiView DAB IHC Detection Kit (Ventana). The mean number of FOXP3 positive stromal cells was determined microscopically per high power field (HPF), counting 3-12 HPF exhibiting the highest histological aGvHD damage.

### Immunofluorescence of Biopsies

FFPE biopsies were cut 2-3 µm thick and were incubated at 80°C for 30 minutes followed by immersing in Xylol twice for 10 minutes each following descending alcohol line for 5 minutes each. Sections blocked with 20% Bovine Serum Albumin (BSA) for 20 min at room temperature (RT). Single immunofluorescence was performed for GPR43 (Biozol, LSA1578-50, rabbit polyclonal). Double immunofluorescence was performed for GPR43 (Biozol, LSA6599, rabbit polyclonal) and CD68 (Dako, PG-M1, mouse monoclonal). Primary antibodies were diluted in 1% BSA and were applied to biopsy section at the dilution of 1:50 for 1 hour at RT followed by secondary antibodies Alexa Flour (AF) 488 and Alexa Flour 594 (Invitrogen) for 30 minutes (dilution 1:100) in the dark at RT. CD68 was conjugated with AF488 and GPR43 was conjugated with AF594. Sections were counterstained with DAPI and were sealed with mounting media. Biopsy sections were washed three times with PBS after every step. GPR43 positive cells were observed and images were taken at 200X magnification using Zeiss epifluorescence microscope.

### Dendritic Cell (DC) Culture and Determination of Cytokines

Monocytes were isolated from PBMC of healthy donors after leukapheresis followed by density gradient centrifugation over Ficoll/Hypaque as described previously (24). All healthy volunteers consented to the study. Freshly isolated monocytes were differentiated into DCs as previously described (17). On day 7, 100 ng/mL LPS (Enzo) was added to induce maturation of immature DCs (iDCs) in absence or presence of 5 mM sodium acetate (NaA), 2.5 mM sodium propionate (NaP) and 0.5 mM sodium butyrate (NaB) for another 48 hours. NaA, NaP and NaB were purchased form Sigma-Aldrich. On day 9, mature DCs (mDCs) were harvested for RNA extraction, cDNA synthesis and qPCR as described above. Supernatants were collected for cytokine determination and were stored at -20°C until further analysis. IL-12 and IL-10 cytokines were analyzed using enzyme-linked immunosorbent assays (ELISA) according to the manufacturer’s recommendation (R&D).

### Caco-2 Cell Culture

The human intestinal Caco-2 cell line was purchased form CLS Germany. Cells were maintained in DMEM low glucose media (Sigma) supplemented with 10% FCS (Sigma), 1% NEAA, 1% NaP and 0.5% P/S (Gibco) in a collagen (5 µg/cm^2^) coated T75 flask. Cells were seeded at 9.3x10^3^ cells/cm^2^ and subcultured after 60-80% confluency for a maximum of 10 passages, changing media every two days. For differentiation, Caco-2 cell monolayer was grown at a density of 3x10^5^ cells/cm^2^ on 0.4 µm collagen coated polyster membrane 12 well transwell (1.12 cm^2^ area) for 3 weeks. The monolayer was monitored by measuring trans epithelial electrical resistance (TEER) with Millicell ERS-2 voltohmmeter (EMD Millipore). Media was changed every two to three days. On day 21, cells were stimulated with 50 ng/ml IL1β and 100 ng/ml TNF (PromoCell) with or without sodium butyrate (Sigma) for 24 hours. Barrier integrity was monitored by TEER measuremernt. Cell supernatants were analysed for IL-6 and IL-8 ELISA as per manufacturer’s recommendation (R&D). Immuofluorescent staining of Caco-2 cells were performed as previously described (17).

### Statistical Analysis

Data analysis was done in SPSS v26. Test of normality was performed using Shapiro-Wilk test. Normally distributed data was analysed with t-test or one way ANOVA. Correlation analysis was performed with Pearson test. For non-normal data, Mann-Whitney or Kruskal Wallis tests were performed and Spearman correlation was chosen. For multivariate analysis, results were dichotomized based on median. The Lerner stage of aGvHD and use of Abx prior to biopsy were analyzed using binary logistic regression.

## Results

### GPR Expression Correlates With the Severity and Onset of GI-aGvHD

**Figure 1:**
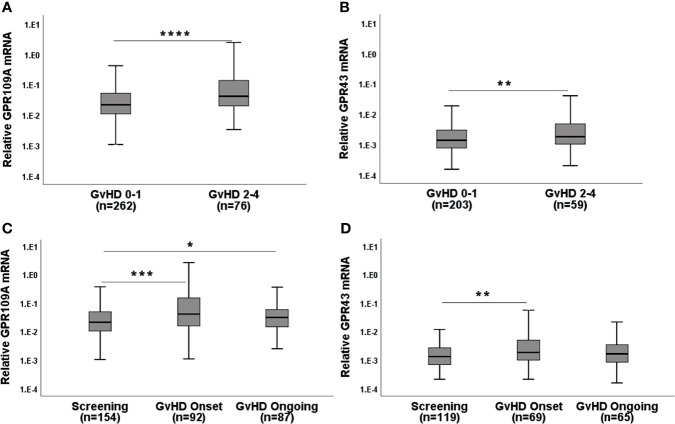
GPR mRNA expression in the serial biopsies from the gastro-intestinal tract in the course of GvHD. **(A)** GPR109A and **(B)** GPR43 expression with respect to Lerner GI-GvHD. **(C)** GPR109A and **(D)** GPR43 expression in screening biopsies and at the clinical onset of GI-GvHD. *p < 0.05, **p < 0.01, ***p < 0.001, ****p < 0.0001, Mann-Whitney U test.

When we assigned unbiasedly selected serial biopsies based on the determined histological Lerner stage to either aGvHD 0-1 or aGvHD 2-4, we found that patients (pts) with higher Lerner stages showed increased GPR expression (**Figures 1A, B**; p=0.000015 for GPR109A, p=0.008 for GPR43). In accordance with this observation, clinical symptomatic aGvHD pts showed higher GPR expression (p=0.0001 for GPR109A, p=0.006 for GPR43) compared to aGvHD free screening pts or ongoing aGvHD pts (**Figures 1C, D**). In addition, the phenomenon of GPR upregulation was observed in both upper or lower gastro-intestinal (GI) tract (**Table 1**). In summary, GPR expression was upregulated in both histological and clinical aGvHD independent of anatomical section of biopsy.

**Table 1:** Distribution of GPR109A and GPR43 in the upper and lower gastro-intestinal (GI) tract.

A. Histological GvHD					
Genes	Lerner stage	No of samples	Mean rank		P value
**Upper Gastrointestinal tract**					
GPR109A	0-1	82	47.2		0.003
	2-4	20	69.15		
GPR43	0-1	70	41.06		0.058
	2-4	16	54.19		
**Lower Gastrointestinal tract**					
GPR109A	0-1	179	110.56		0.002
	2-4	57	143.43		
GPR43	0-1	133	83.96		0.023
	2-4	44	104.43		
**B. Clinical GvHD**					
**Genes**	**Clinical character**	**No of samples**	**Mean rank**		**P value**
**Upper Gastrointestinal tract**					
GPR109A	Screening	51	31.69		0.005
	Onset	20	47.00		
GPR43	Screening	43	27.12		0.017
	Onset	17	39.06		
**Lower Gastrointestinal tract**					
GPR109A	Screening	103	79.37		0.007
	Onset	72	100.34		
GPR43	Screening	76	58.61		0.030
	Onset	52	73.12		

**(A)** GPR distribution in the GI tract according to the Lerner classification of acute GvHD (GvHD 0-1 vs GvHD 2-4). **(B)** GPR distribution in the GI tract according to the clinical characteristics of acute GvHD (screening vs onset).

### Broad Spectrum Antibiotics (Abx) Suppress GPR and FOXP3 Expression

**Figure 2:**
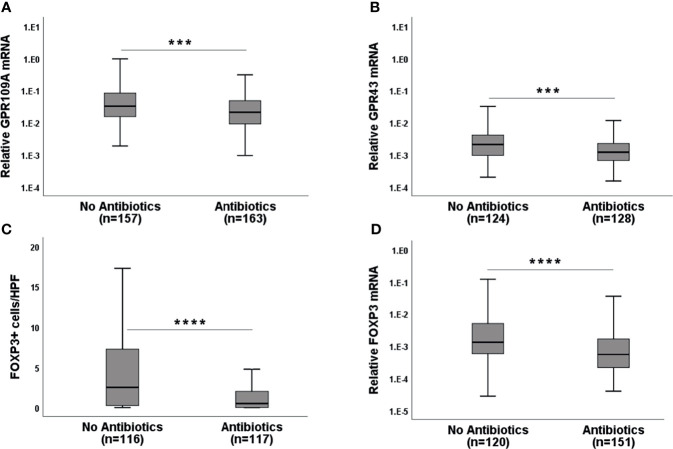
Effect of broad-spectrum antibiotics (Abx) on GPR and FOXP3 expression in the serial biopsies from the gastro-intestinal tract. **(A)** GPR109A mRNA **(B)** GPR43 mRNA **(C)** FOXP3+ cellular infiltrates and **(D)** FOXP3 mRNA expression in the gut biopsies of patients without or with broad-spectrum antibiotic exposure at the time of biopsy retrieval. HPF-high power field. ***p < 0.001, ****p < 0.0001, Mann-Whitney U test.

**Figure 3:**
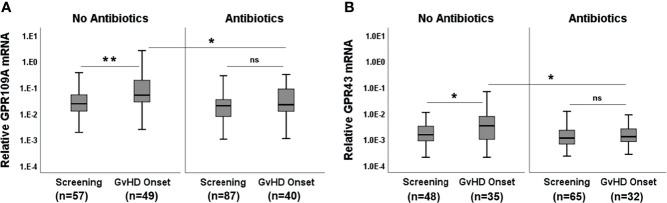
Effect of Abx at the onset of GvHD. **(A)** GPR109A expression at GvHD onset without or with Abx. **(B)** GPR43 expression at GvHD onset without or with Abx. *p < 0.05, **p < 0.01, Mann-Whitney U test. ns, not significant.

Broad spectrum Abx results in rapid loss of microbiota diversity. We, therefore, considered the application of Abx (mainly piperacillin/tazobactam or carbapenems) within 7 days before obtaining biopsies as an indicator of microbiota damage. GPR expression in Abx group was significantly reduced compared to the no Abx group (**Figures 2A, B**, p=0.0004 for GPR109A and p= 0.0001 for GPR43) suggesting that commensal bacteria and their metabolites are needed for optimal GPR induction. Abx suppressed not only GPR, but also FOXP3 mRNA, as well as FOXP3+ regulatory cell (Tregs) infiltrates (**Figures 2C, D**, p<0.0001 forboth FOXP3 mRNA and protein). Following these results, we subsequently classified pts based on cumulative and long-term antibiotic exposure. The first group did not receive early Abx (before or at day 0 of transplantation) or at the time of biopsy. The second group received early Abx but not at the time of biopsy. The third group had Abx at the time of biopsy but no early exposure. The fourth group had both early Abx exposure and at the time of biopsy. The highest GPR expression was observed in the patient group who never had Ab exposure (**Supplementary Figures 1A, B**, p=0.002 for GPR109A, p=0.016 for GPR43, Kruskal-Wallis test). A similar picture was obtained for FOXP3 mRNA and Tregs infiltrates (**Supplementary Figures 1C, D**, p=0.007 for FOXP3+ cellular infiltrates, p=0.0004 for FOXP3 mRNA, Kruskal-Wallis test). Significant loss of GPR was observed in the patient group with early Abx and Abx at biopsy. This may result from previously reported rapid loss of commensals after Abx treatment to pts and is reflected by reduced GPR expression in the gut biopsies. When we grouped pts according to the clinical GI-aGvHD status at the time of biopsies (GI-aGvHD free screening biopsies and aGvHD clinical onset biopsies) and further re-grouped them again according to the use of Abx, our findings were confirmed in the serial biopsies. Pts who did not receive Abx showed significant increases in GPR at the onset of aGvHD (p=0.001 for GPR109A, p=0.014 for GPR43) whereas pts with Abx did not show GPR upregulation at the aGvHD onset (**Figures 3A, B**). Moreover, in the screening biopsies, GPR109A expression was significantly downregulated in the Abx group (p=0.028) whereas GPR43 only showed a trend of downregulation. At aGvHD onset, both GPR showed significant downregulation in the Abx group (p=0.004 for GPR109A, p=0.021 for GPR43) suggesting a detrimental effect of Abx in the course of protective GPR upregulation. When we performed binary logistic regression comparing aGvHD Lerner stage and Abx use, we identified antibiotic use but not aGvHD as an independent risk factor for the loss of GPR as well as FOXP3 (**Supplementary Table 2**).

### Association of GPR With FOXP3 and NLRP3 Expression

**Figure 4:**
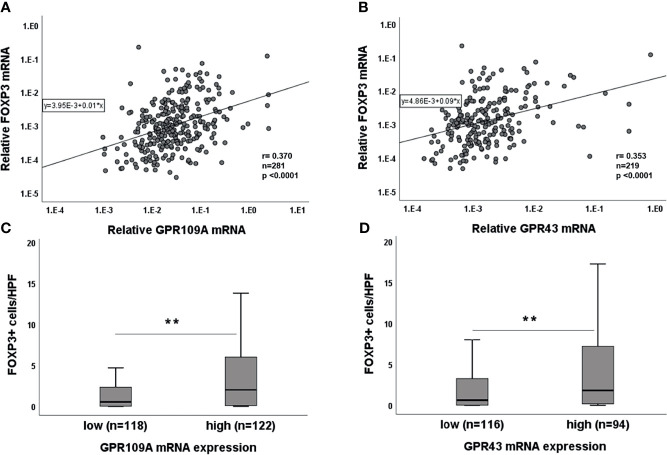
Association of GPR with FOXP3 expression. Correlation of **(A)** GPR109A and **(B)** GPR43 with FOXP3 mRNA. Association of **(C)** GPR109A and **(D)** GPR43 with FOXP3 cellular infiltrates. **p < 0.01, Mann-Whitney U test; r value, Spearman correlation.

As SCFA have been reported to be involved in immunoregulation, we performed simultaneous PCR for FOXP3 expression in the serial gut biopsies. A highly significant correlation between GPR and FOXP3 was observed for both GPR (**Figures 4A, B**, p<0.0001). We dichotomized GPR expressions as “high” and “low” categories based on their median expression (median value: 2.57xE-002 for GPR109A and 1.5xE-003 for GPR43). Higher GPR expression was associated with higher FOXP3 expression and vice versa (p=0.000 for both GPR, data not shown). To confirm this association, we performed immunohistochemistry for FOXP3+ cellular infiltrates. We found that Tregs infiltration was significantly higher in GPR “high” category (p=0.001 for GPR109A, p=0.003 for GPR43) compared to GPR “low” category (**Figures 4C, D**). In addition, binary logistic regression confirmed that both GPR109A and GPR43 independently influence FOXP3 expression (GPR109A: odds ratio,0.74 [95% CI, 1.24-3.55]; p=0.005, GPR43: odds ratio, 0.61 [95% CI, 1.08-3.17]; p=0.024). We also observed a strong association of GPRs with inflammasome receptor NLRP3 in a serial biopsies (**Supplementary Figure 2A, B**). Patient biopsies with high GPR43 expression also had significantly higher NLRP3 expression (p=0.003). GPR109A, although not significant, showed a strong trend of upregulation with higher NLRP3 expression (p=0.087). Regression model revealed that GPR43, but not GPR109A, independently influence NLRP3 expression (GPR43: odds ratio,1.03 [95% CI, 1.1-6.7]; p=0.02, GPR109A: odds ratio, 0.54 [95% CI, 0.71-4.1]; p=0.2). The strong GPR-NLRP3 association was only seen in pts not receiving Abx. Pts on Abx did not show any GPR-NLRP3 association (**Supplementary Figure 2C, D**).

### Epithelial Cells and Immune Cells as a Cellular Source of GPR

**Figure 5:**
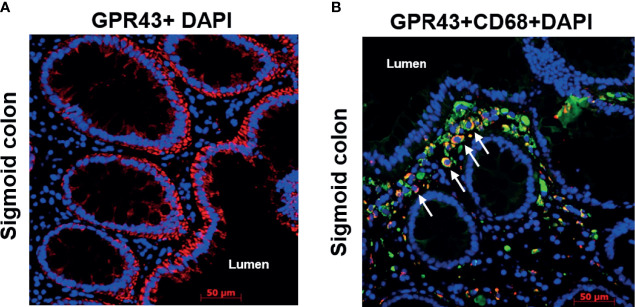
Immunofluorescence of GPR43 of a representative patient biopsy. Time from transplant to biopsy: 3.5 years, no GvHD at the time of biopsy. **(A)** GPR43 staining in the sigmoid colon of a patient. GPR43 is labelled with AlexaFlour (AF) 594 (red). **(B)** GPR43 and CD68 co-staining in the sigmoid colon of a patient. GPR43 is labelled with AF594 (red) and CD68 is labelled with AF488 (green). White arrow indicates colocalized signals. Nucleus is counterstained with DAPI (blue). Scale bar: 50 µm.

To identify the cellular source of GPR, we next performed single and double immunofluorescence of GPR43 and CD68 on sigmoid colon biopsies of pts following transplantation. Within the non-hematopoietic compartment, epithelial cells seemed to be the major source of GPR expression (**Figure 5A**) labeled by GPR43 antibody (cytoplasmic domain, LS-A1578). The gut lumen bears the highest concentration of SCFA and gut epithelium may express GPR in a positive feedback loop. In double immunofluorescence of CD68 and GPR43 (extracellular domain, LS-A6599), two signals co-localized suggesting macrophages as one of the cellular sources of GPR within the immune cell compartment (**Figure 5B**). These GPR43 positive macrophages seemed to accumulate close to the epithelium. The involvement of immune cells in GPR expression is also suggested by the localization dependent expression of GPR. When we compared GPR expression in serial biopsies obtained from different anatomical sections of the gastrointestinal tract, significant higher GPR (p=0.002 for GPR109A, p=0.001 GPR43, Kruskal-Wallis test) was observed in ileal biopsies (**Supplementary Figures 3A, B**). This might reflect a higher presence of immune cells in the ileum.

### Effect of SCFA on Immune Cells and Epithelial Cells *In Vitro*

#### SCFA Upregulate GPR Expression and Alter Cytokine Production in mDCs

**Figure 6:**
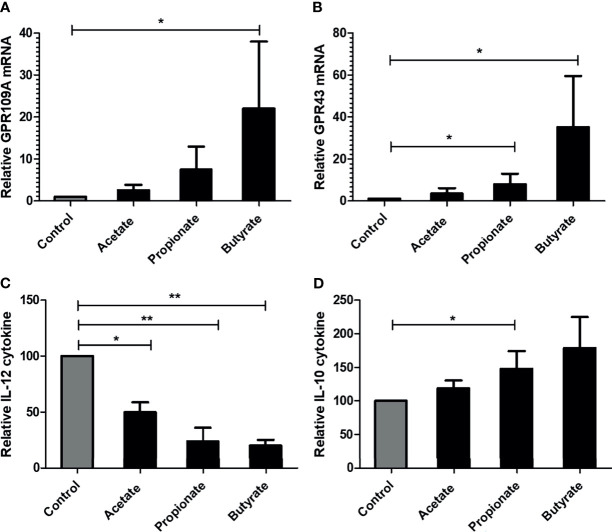
Effect of SCFA on in-vitro generated human monocyte derived DCs. DCs were cultured for 7 days and were stimulated with 100 ng/ml LPS for 48 hours. **(A, B)** GPR109A and GPR43 expression in mature DCs in presence of SCFA. **(C)** IL-12 cytokine release by DCs in presence of SCFA. **(D)** IL-10 cytokine release by DCs in presence of SCFA. n= 3 healthy donors. Bar represents mean +/- s.e.m. *p < 0.05, **p < 0.01, Mann-Whitney U test for A, B and D (non-normal distribution), one way ANOVA with Bonferroni correction for C (normal distribution).

We next assessed the effect of SCFA in lipopolysaccharide (LPS) stimulated monocyte-derived dendritic cells (mDCs) from three healthy donors. 5mM acetate or 2.5 mM propionate or 0.5 mM butyrate was added together with LPS. The given concentration of SCFA did not induce cell death of mDCs when compared to control mDCs as observed by Annexin/7-AAD staining (data not shown). SCFA, especially butyrate, induced significantly higher expression of GPR109A and GPR43 in mDCs (**Figures 6A, B**). At the functional level, SCFAs were able to suppress the LPS induced activation of mDCs indicated by a reduction of pro-inflammatory cytokine IL-12 (**Figure 6C**) and an upregulation of anti-inflammatory cytokine IL-10 (**Figure 6D**).

#### Butyrate Suppresses Pro-Inflammatory Cytokines and Induce GPR43 Expression in Caco-2 Cells

**Figure 7:**
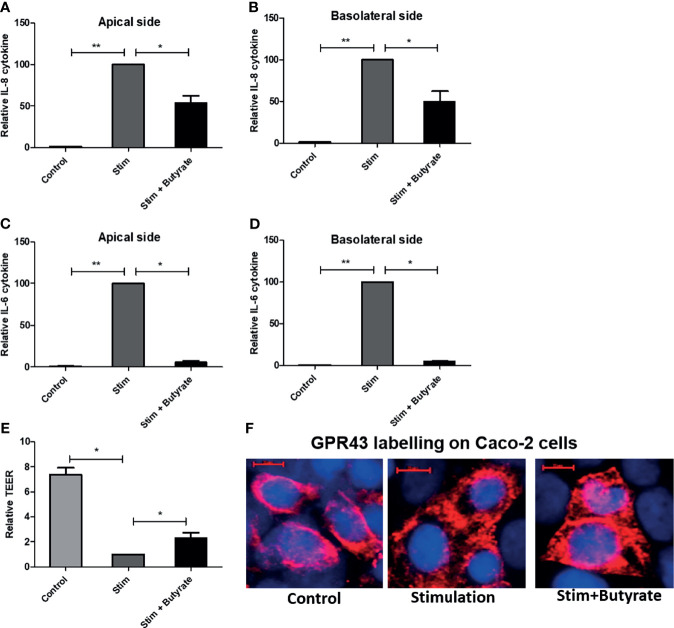
Effect of butyrate on human epithelial cell. Caco-2 cell were grown on collagen coated transwell for 21 days. Cell were treated with IL-1β and TNF for 24 hours without or with butyrate. **(A, B)** IL-8 cytokine release on apical and basolateral side of Caco-2 cells. **(C, D)** IL-6 cytokine release on apical and basolateral side of Caco-2 cells. **(E)**. Transepithelial electrical resistance (TEER) changes with stimulation (stim) alone or with butyrate. **(F)** GPR43 staining of Caco-2 cells in untreated control, stimulated control and butyrate treated condition. GPR43 is labelled with AF 594. Nucleus is counterstained with DAPI. Scale bar: 10µm. n = 4 independent experiments. Bar represents mean +/- s.e.m. *p < 0.05, **p < 0.01, Mann-Whitney U test.

Following the immunomodulatory effect of butyrate on dendritic cells, we sought to investigate the effect of butyrate on epithelial cell line model Caco-2. In four individual experiments, fully differentiated Caco-2 cells on a transwell system were stimulated with 50 ng/ml IL-1β and 100 ng/ml TNF. 5 mM butyrate was added to the stimulated cells for 24 hours. Butyrate toxicity was excluded by MTT assay (data not shown). In absence of stimulation, Caco-2 (control) cells did not produce cytokines. IL-1 β stimulation was a pre- requisite for cytokine production by Caco-2 cells. The production of pro-inflammatory cytokine IL-8 and IL-6 by Caco-2 cells was significantly suppressed on both apical and basolateral side of the transwell system by the addition of butyrate (stim+butyrate) when compared to stimulated condition (stim) (**Figures 7A–D**). Stimulation also compromised barrier intergrity as shown by significant reduction of transepithelial electrical resistance (TEER) when compared to control (**Figure 7E**). The addition of butyrate showed rescue effect by significantly increasing TEER (**Figure 7E**). When we labelled Caco-2 cells with GPR43 antibody, we observed a stronger GPR43 signal in butyrate-treated epithelial cells compared to untreated control or stimulated control (**Figure 7F**).

## Discussion

The human gut harbors a plethora of microorganisms that are crucial for development and normal physiological functions. An imbalance or maladaptations of these essential microorganisms, also termed dysbiosis, has been linked to numerous intestinal disorders including GvHD. Several studies have confirmed a strong association of microbiota damage with the occurrence of GvHD and associated transplant-related complications (7, 25, 26). Microbiota-derived SCFA such as acetate, propionate, and butyrate have been described in previous studies to be the key modulator of inflammation and GvHD by promoting anti-inflammatory myeloid cells and by maintaining epithelial barrier integrity (13, 19, 27, 28). These studies also revealed the involvement of G-protein coupled receptors GPR109A, GPR43, and GPR41 in the mitigation of GvHD. However, these studies were performed in mice and no data have previously been reported regarding the role of GPR expression in adult human GvHD.

Our clinical data show an increased expression of GPR43 and GPR109A in patients (pts) suffering from GvHD. Especially during GvHD onset or at higher grade GI-GvHD (Lerner II-IV), GPR expression was significantly enhanced. This might reflect a counter-regulatory mechanism of protective GPR signaling that is reactively induced to suppress T cell-mediated injury. There are only a few studies discussing counter-regulatory mechanisms in the gut of GvHD pts (29, 30). Landfried et al. showed an increase of IDO in the lower GI tract of GvHD pts (29) while Lord et al. showed an increase of FOXP3 Tregs in the gastric biopsies of GvHD pts (30). Takatsuka et al. showed significant increase of plasma IL-10 in GvHD patients (31). We speculate that the actual increase in regulatory parameters such as IDO, FOXP3, IL-10, and GPR in GvHD is a physiological counter-reaction to suppress the various inflammatory reactions going on in patients’ system. In addition, it is known that inflammatory stimuli such as TNF, IL-1, LPS or GM-CSF can induce GPR expression on monocytes (32) and macrophages (33). Therefore, it is likely that the induction of GPR is, in part, the result of elevated inflammation in GvHD.

Commensal bacteria are the most prominent SCFA producers and have been reported to be suppressed after antibiotic treatment (9, 34). We recently demonstrated that Abx suppresses butyrogeneic bacteria that are responsible for SCFA production (35). Consistently, we found that (i) SCFAs induce GPR expression in human colon cell lines and mDCs and (ii) Abx significantly suppressed GPR expression in the intestinal biopsies of allo-SCT pts. Utilizing a regression model, Abx suppressed GPR expression independent of GvHD. Herein, we propose that the detrimental effect of Abx are confined not only to loss of commensals following reduction of SCFA but also to the loss of GPR expression. Cumulative and long-term antibiotic exposure revealed that GPR expression was highest in pts who did not receive Abx either before/at transplantation or before biopsy retrieval. On contrary, the lowest GPR expression was observed in pts who received Abx before transplantation and also at the time of biopsy indicating persistent long-term dysbiosis by cumulative ABX exposure. The fact that GPR upregulates in GvHD onset pts only in the absence of Abx but not in presence of Abx implicates the potentially protective “commensal-SCFA-GPR” axis in GvHD patients which is clearly abrogated by Abx.

SCFAs have been reported to expand regulatory T cells (15, 36) and these cells prevent GvHD and promote immune reconstitution (37–40). We, therefore, addressed the interrelation of GPR and FOXP3 expression. We observed a high correlation between GPR and FOXP3 expression on mRNA level which was also confirmed in pts where immune cell infiltrates were directly stained for FOXP3 and the positive cells were counted in high power field (HPF). Strong association of GPR with Treg infiltrates point towards the GPR-FOXP3 axis that is again abrogated by the use of Abx. In addition, we saw a negative correlation between Abx use and FOXP3 expression suggesting a link between microbiota changes and immunoregulation although the exact pathways linking ABX use and FOXP3 suppression need to be further analyzed. As we used single antibody staining for our immunohistological analysis of FOXP3+ cells, we are thus far unable to further characterize the Treg subpopulations in more detail. In the clinical setting, it is still unclear whether both natural and induced Tregs are affected by SCFA and future studies using multiplex staining are required to address these questions.

Previous murine studies reported that the salutary effect of GPR in mitigating GvHD occurred *via* non-hematopoietic cells, namely intestinal epithelial cells in an NLRP3 dependent fashion (19, 20). In line with murine data, we also observed a strong association of NLRP3 with GPR expression in patient gut biopsies supporting the GPR regulation in epithelial cells. Immunofluorescence revealed intestinal epithelial cells as one of the cellular sources of GPR43 within the non-hematopoietic compartment which is in line with a previous study (41). In an intestinal epithelial cell line model, butyrate suppressed inflammatory cytokine release, rescued the damaged epithelial barrier and increased GPR43 expression indicating the positive feedback loop of ligand-receptor interaction. Within the hematopoietic compartment, CD68 positive macrophages coexpressed GPR43. Previous murine and human studies described leukocyte subpopulation as a source of GPR43 (42, 43). Immune cells like macrophages, dendritic cells, monocytes, and neutrophils likely play an inevitable role in GPR-mediated protection from GvHD and antibiotic treatment abrogates the necessary protective phenomenon due to dysbiosis, or, by inhibiting the bacterial translocation that would otherwise induce immune responses. Upon treatment with SCFA, in-vitro generated mDCs showed increased expression of GPR109A and GPR43 followed by reduced pro-inflammatory IL-12 and an increase in anti-inflammatory IL-10 cytokine release pointing towards the immunoregulatory phenomenon of SCFA and are in line with previous reports where bacterial metabolite exerted immune regulation by modulating antigen-presenting cells (17, 44). In our study, pts showed higher expression of both GPR in the ileum and there was a gradual recovery of GPR over the time after transplantation implicating the role of hematopoietic cells and recovering epithelial tissue. Our data is in line with previous murine studies that reported the involvement of immune cells in GPR-mediated protection against inflammation (27, 45).

Our study has some limitations. We were not able to directly assess microbiome status at the time of biopsy retrieval. This limited simultaneous analysis of GPR expression and microbial diversity and prompted us to use antibiotic treatment as a surrogate in the clinical settings of GvHD. Furthermore, epithelial interactions of SCFA with GPR are likely to be directly influenced by luminal metabolites of commensals, however, we do not know yet about the exact role of translocated bacteria and tissue metabolites which are likely to play an additional role due to the leakiness of epithelia in GvHD and tissue immune regulation (46). Nevertheless, our study is the first to address the interaction of microbiota and regulation of adaptive immune responses in human tissue biopsies of SCT pts. So far, only stimulation of peripheral blood Tregs has been reported in pts receiving fecal microbiota transplant (FMT) from healthy donors for treatment of refractory GvHD (11), thus both observations point to the fact that a diverse microbiota is needed to mount an adequate Treg cell response. Whether the observed association of GPR and FOXP3 expression is due to a direct effect of SCFA on Tregs induction, e.g. *via* HDAC inhibition as reported in an earlier study (15), or involves further mediators released by immune or epithelial cells, needs further investigation. The negative impact of antibiotic treatment on Treg cell response in tissues has so far been reported outside HSCT models. An association of early-life antibiotic exposure and the development of experimental asthma in murine models have been observed (47). In a murine model of pulmonary metastases, antibiotic treatment reduced T regs and increased the cytotoxic T cell response (48). Similarly, experimental FMT has been shown to increase Treg cell frequencies in the gut which were diminished after Abx exposure (49, 50). Overall, our observations are in line with the protective effects of high SCFA producing commensals in HSCT-associated complications and support the concept that microbiota restoration, e.g. by FMT may be beneficial in GvHD pts. So far, only a small and casuistic series of successful FMTs in clinical GvHD has been reported, but thoroughly designed clinical trials are now initiated to examine the exact contribution of microbiota reconstitution by FMT or more specific consortia of commensals to immunomodulation of GvHD.

To conclude, our data suggest so far neglected but deleterious effects of Abx on GPR expression and immunoregulation in clinical GvHD. We urge the need for microbiota preservation or restoration either by FMT, transfer of protective commensal consortia or by fiber-rich diet (51). In addition, our data strongly suggest restrictive use of Abx and support careful antibiotic stewardship to maintain microbiota, metabolites, receptors, and immunoregulation. This approach might be relevant for GvHD prophylaxis and treatment as well as several other diseases where dysbiosis is concerned.

## Data Availability Statement

The original contributions presented in the study are included in the article/**Supplementary Material**. Further inquiries can be directed to the corresponding authors.

## Ethics Statement

The studies involving human participants were reviewed and approved by Aktive Ethikvoten der Ethik-Kommission an der Universität Regensburg Email: ethikkomission@ur.de. The patients/participants provided their written informed consent to participate in this study (approval no: 02/220, 09/059, 17-619-101).

## Author Contributions

SakG performed the experiments, collected and analyzed the data, and wrote the manuscript. DWe designed the study, analyzed clinical data and discussed the manuscript. KH performed immunohistological analyses. EM, MH, and AS-K collected and analyzed clinical data in relation to biopsy results. AG, AH, CM, SarG, DWo, ME, PH, HP, and WH reviewed and discussed the manuscript. EH designed the study, performed data analysis and wrote the manuscript. All authors contributed to the article and approved the submitted version.

## Funding

This work was supported by the Wilhelm Sander Foundation, Grant 2017.020.1 “Dysbiosis and intestinal immunoregulation in GvHD”. This work was partially funded by the Deutsche Forschungsgemeinschaft (DFG, German Research Foundation) - Projektnummer 324392634 - TRR 221”, partially by Marie Curie Initial Training Networks, Project Number 315963, partially by Mechtild Harf Research Grant from the DKMS Foundation for Giving Life, and by the Else-Kroener-Fresenius-Stiftung. DKMS Foundation for Giving Life, the Else-Kroener-Fresenius-Stiftung, CRC 1371, DFG “Microbiome signatures”.

## Conflict of Interest

The authors declare that the research was conducted in the absence of any commercial or financial relationships that could be construed as a potential conflict of interest.

## Publisher’s Note

All claims expressed in this article are solely those of the authors and do not necessarily represent those of their affiliated organizations, or those of the publisher, the editors and the reviewers. Any product that may be evaluated in this article, or claim that may be made by its manufacturer, is not guaranteed or endorsed by the publisher.

## Supplementary Material

Supplementary Table 1 Clinical characteristics of patients: MDS = myelodysplastic syndrome, MPS = myeloproliferative syndrome, BM, bone marrow; PBSC, peripheral blood stem cells; UCB, umbilical cord blood; CyA, cyclosporine; MTX, Methotrexate; Tacro, tacrolimus; MMF, mycophenolate mofetil; PTCy, post transplant cyclophosphamide.

Supplementary Table 2 Analysis of factors influencing GPRs and FOXP3 expression. In a multivariable analysis, Abx use is an independent factor to suppress GPR expression as well as FOXP3 expression. OR, odds ratio; CI, confidence interval.

Supplementary Figure 1 Cumulative effect of broad spectrum antibiotics on the expression of **(A)** GPR109A, **(B)** GPR43, **(C)** FOXP3+ cellular infiltrates and **(D)** FOXP3 mRNA. *p < 0.05, **p < 0.01, ***p < 0.001, Mann-Whitney U test.

Supplementary Figure 2 Association of **(A)** GPR109A and **(B)** GPR43 with NLRP3 expression. Effect of broad-spectrum antibiotics on **(C)** GPR43-NLRP3 association and **(D)** GPR109A-NLRP3 association *p < 0.05, **p < 0.01, ***p < 0.001, Mann-Whitney U test.

Supplementary Figure 3 Distribution of **(A)** GPR109A and **(B)** GPR43 mRNA expression within the GI tract of patients after allogeneic SCT. Stomach, duodenum, ileum, colon and sigmoid colon were evaluated in the serial biopsies of transplanted patients. *p < 0.05, **p < 0.01, ***p < 0.001, Mann-Whitney U test.
