# Supplementary material for: GPR Expression in Intestinal Biopsies From SCT Patients Is Upregulated in GvHD and Is Suppressed by Broad-Spectrum Antibiotics
Source: Front Immunol. 2021 Oct 28;12:753287. doi: 10.3389/fimmu.2021.753287 (PMC8588834; doi:10.3389/fimmu.2021.753287)
Supplement: Supplementary Table 1 — Clinical characteristics of patients: MDS = myelodysplastic syndrome, MPS = myeloproliferative syndrome, BM, bone marrow; PBSC, peripheral blood stem cells; UCB, umbilical cord blood; CyA, cyclosporine; MTX, Methotrexate; Tacro, tacrolimus; MMF, mycophenolate mofetil; PTCy, post transplant cyclophosphamide. [file Table_1.docx]

|  |  | **mean** | **(range)** |
| --- | --- | --- | --- |
| **Age** | in years | 51,9 | (19,2-70,7) |
|  |  | n | (%) |
| **Sex** | male | 124 | 62,3 |
|  | female | 75 | 37,7 |
| **Diagnosis** | Acute leukemia | 108 | 54,3 |
|  | MDS/MPS | 31 | 15,6 |
|  | Lymphoma | 55 | 27,6 |
|  | Aplastic Anemia | 4 | 2,0 |
| **Disease stage** | early | 43 | 21,6 |
|  | intermediate | 76 | 38,2 |
|  | advanced | 80 | 40,2 |
| **Donor type** | Unrelated donor | 128 | 64,3 |
|  | Sibling | 65 | 32,7 |
|  | Haploidentical donor | 6 | 3,0 |
| **Stem cell source** | PBSC | 182 | 91,5 |
|  | BM | 16 | 8,0 |
|  | UCB | 1 | 0,5 |
| **Conditioning regimen** | Reduced intensity | 173 | 86,9 |
|  | Standard | 26 | 13,1 |
| **GvHD Prophylaxis** | CyA/MTX | 167 | 83,9 |
|  | CyA/MMF | 21 | 10,6 |
|  | Everolimus | 1 | 0,5 |
|  | Tacro/MTX | 2 | 1,0 |
|  | Tacro/MMF | 2 | 1,0 |
|  | PTCy/Tacro/MMF | 6 | 3,0 |
