# Supplementary material for: GPR Expression in Intestinal Biopsies From SCT Patients Is Upregulated in GvHD and Is Suppressed by Broad-Spectrum Antibiotics
Source: Front Immunol. 2021 Oct 28;12:753287. doi: 10.3389/fimmu.2021.753287 (PMC8588834; doi:10.3389/fimmu.2021.753287)
Supplement: Supplementary Table 2 — Analysis of factors influencing GPRs and FOXP3 expression. In a multivariable analysis, Abx use is an independent factor to suppress GPR expression as well as FOXP3 expression. OR, odds ratio; CI, confidence interval. [file Table_2.docx]

| Genes | Risk factor | P value | OR | 95% CI |
| --- | --- | --- | --- | --- |
| GPR109A | GvHD grade **(0-1 vs 2-4)** | 0.3 | 0.991 | 0.976 - 1.008 |
|  | Antibiotics use **(no vs yes)** | 0.001 | 0.475 | 0.304 - 0.744 |
| GPR43 | GvHD grade **(0-1 vs 2-4)** | 0.059 | 0.985 | 0.969 - 1.001 |
|  | Antibiotics use **(no vs yes)** | 0.006 | 0.509 | 0.313 - 0.828 |
| FOXP3 | GvHD grade **(0-1 vs 2-4)** | 0.056 | 1.818 | 0.984 - 3.358 |
|  | Antibiotics use **(no vs yes)** | 0.001 | 0.425 | 0.257 - 0.701 |
| FOXP3 protein (cellular infiltrates) | GvHD grade **0-1 vs 2-4)** | 0.567 | 0.996 | 0.981 - 1.011 |
|  | Antibiotics use **(no vs yes)** | 0.000 | 0.265 | 0.153 - 0.460 |
