# Supplementary figures and images for: GPR Expression in Intestinal Biopsies From SCT Patients Is Upregulated in GvHD and Is Suppressed by Broad-Spectrum Antibiotics
Source: Front Immunol. 2021 Oct 28;12:753287. doi: 10.3389/fimmu.2021.753287 (PMC8588834; doi:10.3389/fimmu.2021.753287)

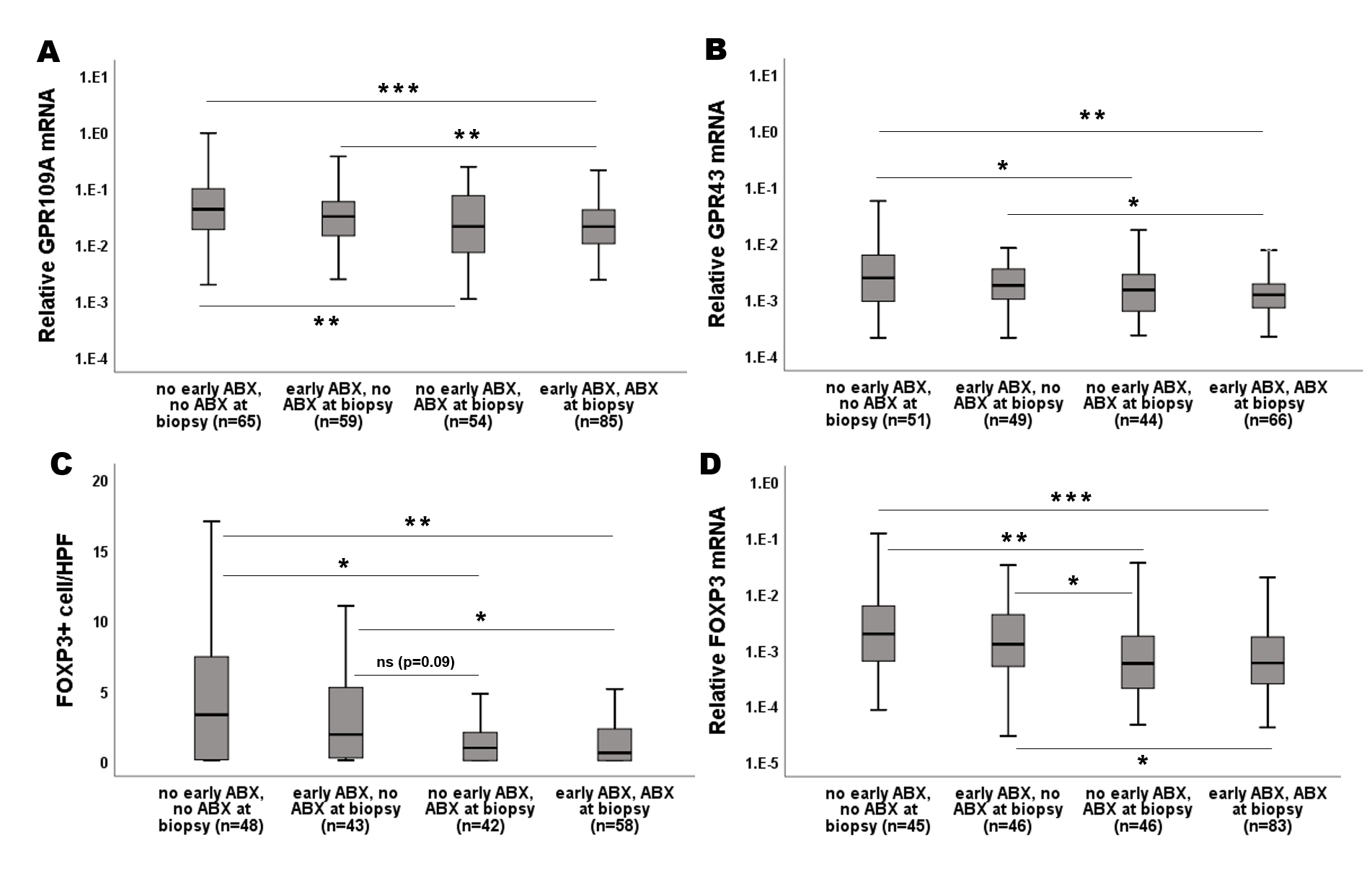

Supplement: Supplementary Figure 1 — Cumulative effect of broad spectrum antibiotics on the expression of (A) GPR109A, (B) GPR43, (C) FOXP3+ cellular infiltrates and (D) FOXP3 mRNA. *p < 0.05, **p < 0.01, ***p < 0.001, Mann-Whitney U test. [file Image_1.tif]

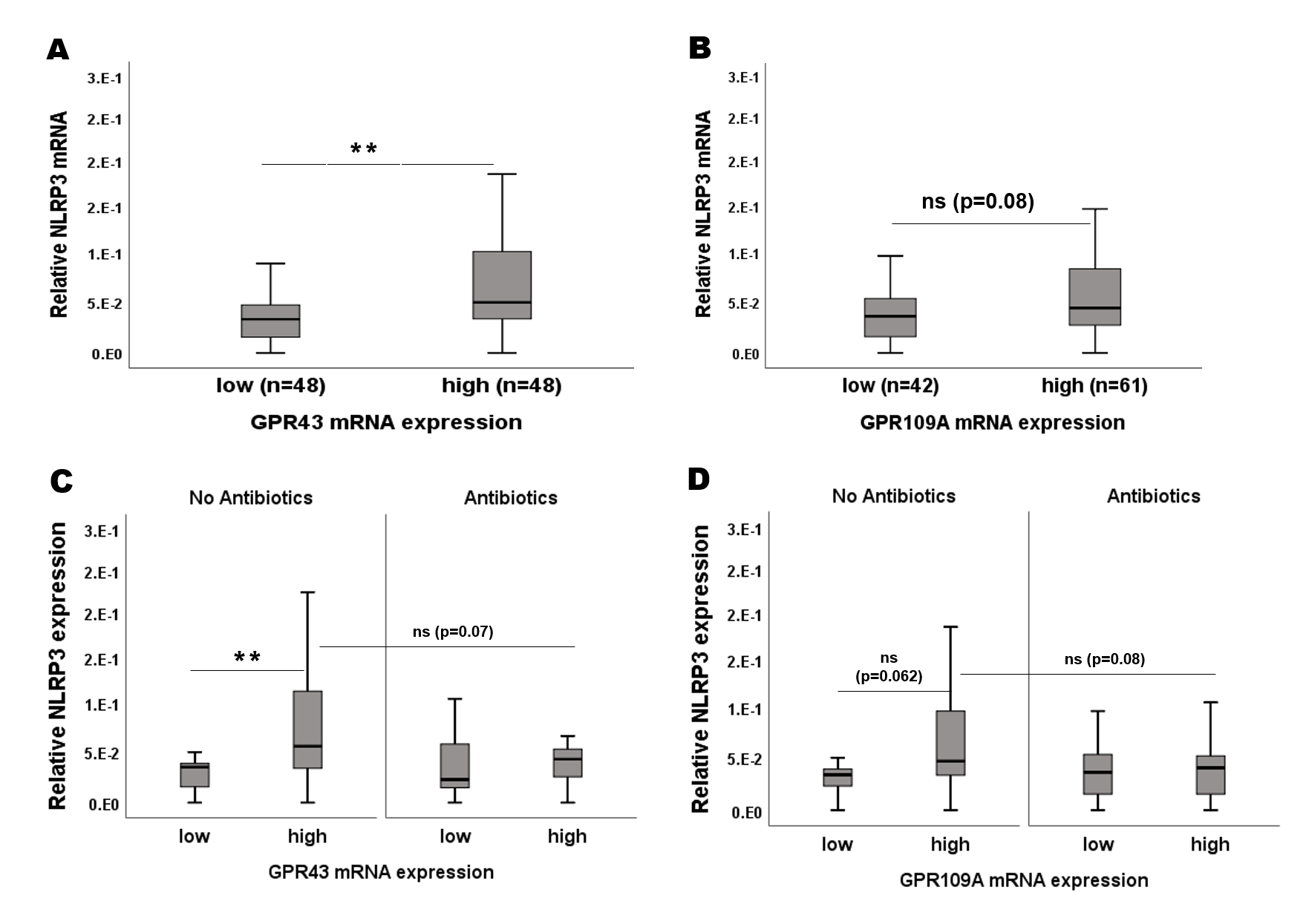

Supplement: Supplementary Figure 2 — Association of (A) GPR109A and (B) GPR43 with NLRP3 expression. Effect of broad-spectrum antibiotics on (C) GPR43-NLRP3 association and (D) GPR109A-NLRP3 association *p < 0.05, **p < 0.01, ***p < 0.001, Mann-Whitney U test. [file Image_2.tif]

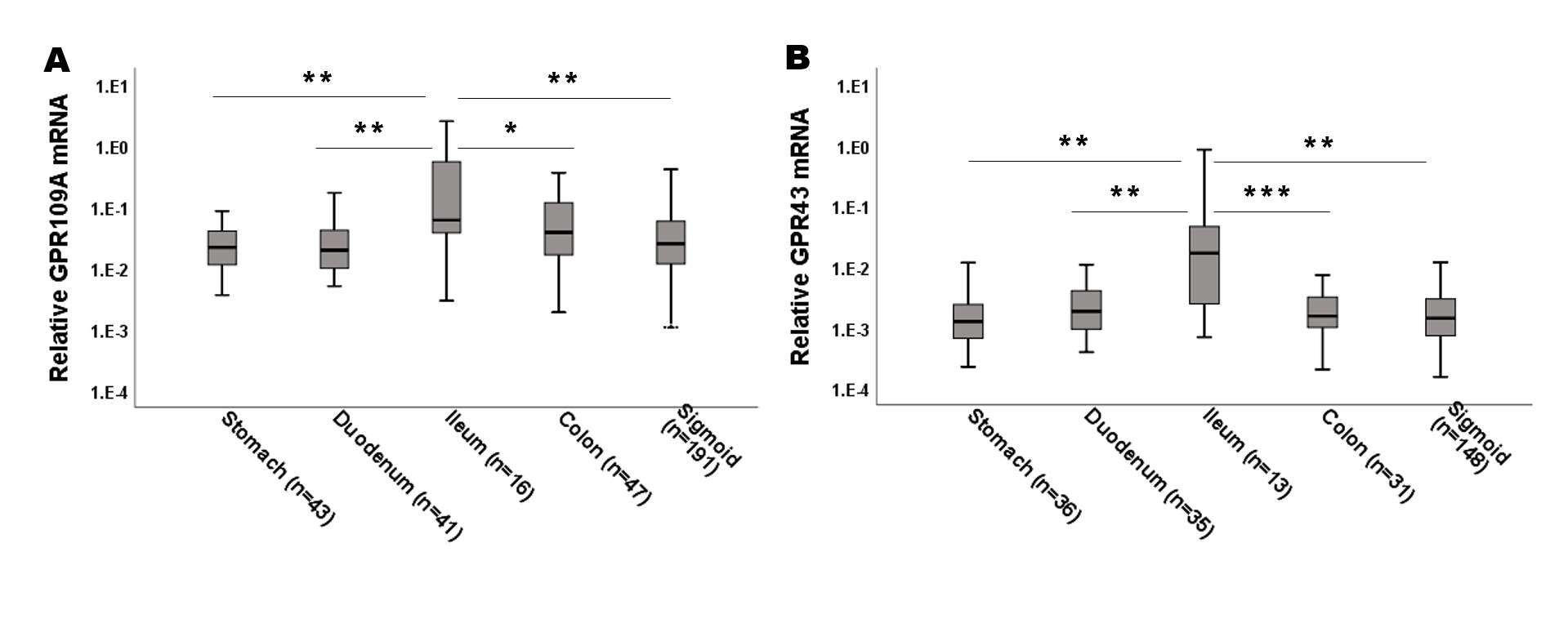

Supplement: Supplementary Figure 3 — Distribution of (A) GPR109A and (B) GPR43 mRNA expression within the GI tract of patients after allogeneic SCT. Stomach, duodenum, ileum, colon and sigmoid colon were evaluated in the serial biopsies of transplanted patients. *p < 0.05, **p < 0.01, ***p < 0.001, Mann-Whitney U test. [file Image_3.tif]
